# Philadelphia Academy of Surgery

**Published:** 1893-09

**Authors:** 


### PHILADELPHIA ACADEMY OF SURGERY.

PHILADELPHIA, APRIL 1, 1893.

The President, Dr. William Hunt, in the chair.

Dr. John Ashhurst, Jr., read the following paper on

### INDIVIDUAL EXPERIENCE IN THE TREATMENT OF VESICAL CALCULUS.

I find in looking over my records that I have removed calculi from the human body in fifty-one cases. One case was that of a female child, on whom I performed lithectasy, or rapid dilatation of the urethra, but the remaining fifty were in male subjects. In thirty-five of these fifty cases the patients were operated on by lateral lithotomy, which is the cutting operation that I prefer. I recognize that there are cases in which the median operation is to be preferred, and that there are other cases in which the supra-pubic operation is the best, but where the surgeon has the choice of operation, I think that he should select lateral lithotomy. Of the thirty-five cases operated on by the lateral method, twenty were in children under the age of puberty, and in every case the patient recovered. In males beyond the age of puberty, including a fair proportion of quite old persons, I have had fifteen cases with three deaths, but only one of these three was really the result of the operation. That occurred in a case operated on in a neighboring town this winter. Secondary hemorrhage occurred on the ninth or tenth day, and the attempts made by the attending physician to control it were not successful.

I have six cases of the median operation, with one death, to report. In one case the operation was done for the removal of a foreign body, the end of a catheter. In this case I succeeded not only in removing the foreign body, on which there was a small calcareous deposit, but also in relieving the chronic retention of urine, from which the patient had long suffered, by tearing off the median lobe of the prostate with the forceps. This was fully ten years ago; the patient is still living, and I believe has not had occasion to use a catheter since. The case which proved fatal was in a patient in the last stages of cystitis and chronic renal disease, and in which the presence of the stone was simply a complication. An interesting feature in this case was that, in addition to the presence of a stone, there was a large quantity of that semi-organized material which has been described by Vandyke Carter as the animal basis of calculi.

I have one case of the suprapubic operation, in which the stone was a small one, this particular operation being chosen because the case was really one of villous tumor of the bladder, and the presence of the stone was simply a complication. The patient was in a critical condition from hemorrhage at the time of the operation, but made a good recovery.

I have no case of the old-fashioned lithotrity. The operation had already come to be rarely practiced before I had occasion to resort to the crushing method. The early portion of my practice was largely with children, and Bigelow's modification had already become the operation of preference when I first felt I had a case adapted to its performance. I have performed this operation eight times, with six satisfactory recoveries and two deaths. Both the deaths were from uremia, dependent upon chronic disease of the kidney.

I have brought here a number of the calculi which I have removed. The largest weighs three ounces and some drachms. It was removed by the ordinary lateral operation. It was not necessary to enlarge the wound by dividing the right side of the prostate, nor was it necessary to crush the stone. By making a large external wound, by grasping the stone with sufficiently powerful forceps, and by patience in manipulation, this stone was removed without difficulty, and the patient made an excellent convalescence.

The largest number of stones which I have removed from one patient is fifty-four. These were removed by lateral lithotomy. The patient made a good recovery, but returned in a year or so with recurrence of the symptoms from a descent of more stones from the kidney. On that occasion I determined to perform the operation of litholapaxy. The patient did pretty well for a few days, but then the urine became turbid, containing a large quantity of ropy mucus and pus, uremia developed, and the patient died in convulsions. This was a forcible illustration of the risk attending litholapaxy in cases of cystitis, and since the occurrence of that case I make it a rule, where the patient presents cystitis in an advanced degree, to recommend the cutting rather than the crushing operation.

With regard to the results that I have reached from my own experience, I would say, in the first place, that I have never seen any reason to wish for a better operation than lateral lithotomy in children. Litholapaxy has been resorted to successfully a number of times, and with the improved instruments which we now have the operation is a feasible one, while it could hardly be considered such a few years ago. Until within a short time it has not been possible to get instruments of sufficient strength and delicacy for use in the urethræ and bladders of children. Even now, the operation of litholapaxy in children seems to me to be a more severe one than lithotomy. The results of cutting for stone in children are so satisfactory that I think we want nothing better. The great advantage of litholapaxy it seems to me is the short time required for after-treatment. If all goes well, litholapaxy will allow the patient to go about his business in five or six days. This is a great advantage in adults who are engaged in active business; but in young children it is a matter of no importance. At the same time I am willing to admit that the operation has been improved to such an extent that it is one which may be legitimately resorted to in children if the surgeon thinks that it is preferable.

The median operation seems to me to have a very limited field. Cases of foreign body in the bladder, and cases of very small stone, are those to which this operation is adapted. In some of my cases the operation was not begun with the knowledge that a stone was present, but for retention of urine where it was not possible to pass an instrument by the urethra. The argument which has been advanced in favor of this operation, that it is attended with less risk of hemorrhage, does not seem to be entirely well founded. There is very little more risk in the lateral operation. The transverse perineal artery is divided, but with a little care it is not likely that the internal pudic or the artery of the bulb will be injured. In the old days of operation without an anesthetic, it was quite possible that one of these arteries might be wounded in the struggles of the patient. The artery of the bulb can be avoided by striking the staff as far back as possible. The hemorrhage from which I have had trouble has been from the prostatic plexus of veins, and this is quite as likely to occur in the median as in the lateral operation, and, indeed, I have seen very profuse hemorrhage from this source after median section.

The suprapubic operation, although just at present the fashionable method, I should reserve for very large stones, or for cases in which there was some complication, such as tumor, in addition to the stone. Cases of vesical tumor are more satisfactorily dealt with through the suprapubic incision, but where the case is an uncomplicated one of stone, I have not seen any reason to prefer this to the lateral method.

In the female, the operation of lithectasy or rapid dilatation is the one to be chosen, and in almost all cases will be sufficient. Mr. Bryant has shown that stones of considerable size can be removed by this method. In children, stones up to half an inch in diameter can be thus removed. If the stone is larger, it can be broken into several fragments before removal. I believe that the results of this method will be more satisfactory than if an attempt is made to remove the calculus by litholapaxy or by any form of lithotomy. The vesico-vaginal section may leave a permanent fistula. The high operation may, of course, be required for very large stones.

As regards the operation of lateral lithotomy, the points which are to be observed are, in the first place, to make a large external wound. I have seen very serious trouble result from too small an external incision. There is no objection to a large wound through the skin and superficial fascia; if hemorrhage occurs, it is easier to deal with it through a large wound, and drainage is more satisfactorily effected. In the second place, I think that it is of great importance to strike the staff as far back as possible. Instead of striking it where it is most superficial, I endeavor to get as far back toward the horizontal portion of the staff as possible. In that way you avoid wounding the artery of the bulb, and obtain plenty of room where it is needed. My preference is to have the staff firmly hooked up under the pubis, instead of having it made to project in the perineum. I believe that in this way it is more firmly held, and that the surgeon can fix the position of the anatomical points better, and therefore cut with more precision. Having struck the staff, I think, following the advice of Sir William Fergusson, that the deep incision should be made small. I believe that there is a decided advantage in this plan. I do not say that the surgeon should not make the wound in some degree proportionate to the size of the calculus, and in cases where there is a large stone, I am in the habit, as I withdraw the knife, of bringing it slightly away from the staff so as to enlarge the deep wound. In children the knife should be withdrawn in close contact with the staff; but in the adult I drop the knife a little, so as to enlarge the wound in the prostate. The finger is then introduced, and the prostatic enlargement completed by dilatation. I do not at all agree with the view of Mr. Teevan, that it is safer to cut the prostate than to stretch it. In the introduction of the finger, I lay stress on its introduction above the curve of the staff. In children this is very important, for if it is not done the finger may not enter the bladder, but may pass into the recto-vesical space. The surgeon cannot miss the bladder if he passes the finger above the staff, as it is well held up under the pubis.

In my earlier operations I had a great fancy for the scoop in removing calculi, using it as the obstetrician uses the vectis, getting the scoop behind the stone and the finger in front of it, and bringing all out together. Of late years I have used the forceps more and the scoop less, although at times it answers a useful purpose. In the withdrawal of the stone, a mistake that I have often seen made is in not carrying the forceps far enough backward toward the coccyx. The portion of the wound where there is plenty of room is far back. I have seen surgeons try unsuccessfully to remove the stone through the anterior portion of the wound, when it could have been readily removed if the forceps had been dropped toward the back.

In the high operation, it is a great advantage to have the bladder and the rectum distended, though perhaps, not absolutely necessary. There is an advantage too, in lateral lithotomy, in having a moderate quantity of fluid, say about four ounces, in the bladder before the operation, as the gush of water, when the bladder is opened, will bring the stone down on the end of the finger. If, however, the bladder is intolerant, I do not care to have it much distended.

With regard to the operation of litholapaxy, the points which I consider to be of importance, are, in the first place, to crush the stone as thoroughly as can be done, and then, when using the evacuator, to make the stream enter with great gentleness. I believe that cystitis may be aggravated or even caused by using too much force. As regards the rapidity of the operation of litholapaxy, I have no doubt that an operator will do it with greater rapidity as he does it oftener, but for my own part, I have found it a slow operation. I think that no surgeon should undertake it who is not prepared to give as many hours to it as may be necessary. I can recall three cases in the practice of other surgeons in which the patients died as the direct result of having a stone left half crushed in the bladder. Violent cystitis came on and the patients succumbed. Where the operation is undertaken, it should be completed. If the surgeon is not prepared to remove the entire stone at one sitting, he should not undertake the operation at all. This is the operation for small stones in patients with healthy bladders. Cystitis is the most dangerous condition in which to resort to litholapaxy. In the case of an adult presenting himself with stone, my first thought is of litholapaxy. I then consider the various circumstances in the case. Litholapaxy has so many advantages in cases to which it is adapted, that I think it should be the surgeon's first choice.

With regard to the objection that lateral lithotomy may render the patient sterile, I do not see why that should be, provided that the operation is confined to one side of the perineum, and that no undue amount of inflammation follows. If there were a great deal of inflammation, it is quite possible that there might be such obstruction of the *vas deferens* as to prevent the patient from generating with the testis of that side, but there is no more reason why the patient should be rendered sterile by the operation of lateral lithotomy than by the removal of one testicle. In the immense number of operations performed in former years, we never heard of this objection, and I believe that it is rather theoretical than practical.

I have had one case of stone weighing less than two grains, which I diagnosed by the sound, and removed by lateral lithotomy. The patient was a lad who had symptoms of stone in the bladder, and in addition, frequent attacks of sudden and complete retention of urine, due to the calculus entering and plugging the internal meatus. The straining was so excessive that, in the effort to pass water the night before the operation, the patient ruptured subconjunctival vessels in both eyes.

I wish to refer to a few cases of cystotomy from other causes than calculus. I do not include cases where I have operated by Sir Henry Thompson's method of puncturing a contracted bladder, above the pubis. I find that I have opened the bladder by cystotomy in eight cases, six of these being cases of cystitis. Of these six, four recovered, and two died, as the result of the diseased state of the urinary organs. In two instances I have opened the bladder for intense pain in the act of micturition, due to a fissure at the neck of the organ. Both patients recovered. In one case the fissure followed cystitis, the result of gonorrhea, and in the other case the symptoms came on after the use of very large sounds.

I have had one case of cystotomy in a child for tuberculous disease of the bladder. This case was of a good deal of interest. The patient had, at one time, been under the care of the late Professor S. D. Gross, who had sounded the child, and said that he felt a stone. It is to be observed, however, that he never appointed a time to operate, so that it is possible that he may have had some doubts as to the diagnosis. A curious feature of the case was that the father, who was a man of considerable intelligence, declared that he had himself distinctly heard the click of the stone against the instrument. I sounded the child, but was not entirely satisfied that a calculus was present, although, from the history, I thought it probable. The child had all the usual symptoms of stone, except sudden arrest of urine. I asked Dr. Forbes to see the case with me, and we thought it right to open the bladder. No stone was found, but there were discharged twenty or thirty little bodies which I presume were what the older surgeons would have spoken of as fibrinous calculi. They looked like little pieces of catgut. Whether these were masses of tuberculous material, or of inspissated mucus and lymph, I do not know. The patient was relieved of his symptoms, but died two months afterward of tuberculous disease of the mesenteric glands.

